# The Impact of General Self-Efficacy on Psychological Resilience During the COVID-19 Pandemic: The Mediating Role of Posttraumatic Growth and the Moderating Role of Deliberate Rumination

**DOI:** 10.3389/fpsyg.2021.684354

**Published:** 2021-06-23

**Authors:** Wei Zeng, Xingrou Wu, Yanhua Xu, Jiamin Wu, Yuqing Zeng, Jinlian Shao, Dongtao Huang, Ziqi Zhu

**Affiliations:** ^1^School of Geography, South China Normal University, Guangzhou, China; ^2^College of Resource Environment and Tourism, Capital Normal University, Beijing, China; ^3^Office of International Cooperation and Exchange, Dongguan University of Technology, Dongguan, China

**Keywords:** general self-efficacy, psychological resilience, post-traumatic growth, deliberate rumination, moderated mediation, COVID-19

## Abstract

**Purpose:** This study used a moderated mediation model to explore the relationship between general self-efficacy (GSE) and psychological resilience (PR) and the associated mechanisms, the mediating role of posttraumatic growth (PTG), and the moderating role of deliberate rumination (DR) during the coronavirus disease 2019 (COVID-19) pandemic. Knowledge of the relationship between these four variables examined further understanding of the PR improvement mechanism of college students and even the general public.

**Methods:** The college students who participated in this study came from an independent college in Guangdong Province, China. A total of 918 college students completed the survey, and the final data sample size was 881. SPSS 23.0 and PROCESS (version 3.3) were used to conduct Pearson's correlation analysis and hierarchical regression linear analysis on the data.

**Results:** (1) The correlation analysis showed that GSE and PR were positively correlated and that PTG was positively correlated with GSE and PR. DR was positively correlated with GSE, PTG, and PR. (2) The results of mediation analysis showed that GSE had a direct predictive effect on DR, and PTG partially mediated the relationship between the two. (3) The results of moderating effect analysis showed that DR hindered the effect of GSE on PTG but enhanced the positive impact of PTG on PR.

**Conclusions:** General self-efficacy can improve PR under the mediating influence of PTG. DR played a positive moderating role in the relationship between GSE and PTG, and played a negative moderating role in the relationship between PTG and PR. These results advance the understanding of the mechanism between GSE and PR.

## Introduction

The rapid development of the coronavirus disease 2019 (COVID-19) pandemic since December 2019 has threatened physical and mental health (Gismero-Gonzalez et al., 2020; Kang et al., 2020), and this international public health emergency has caused concern around the world. China's epidemic prevention and control program used isolation in the home and mask wearing as the core methods to reduce the spread of the virus between persons. During the most intense period of COVID-19 spread, these prevention and control measures caused great changes in the lives and studies of college students (Wang et al., 2020), who also received a large amount of pandemic information through various media platforms (Chang et al., 2020).

These conditions profoundly affected the mental health of such students (Hou et al., 2021). A number of recent studies have suggested that during the pandemic, the incidence of anxiety was 26.60% and the incidence of depression 21.16% (Chang et al., 2020), rates much higher than observed in surveys carried out under ordinary conditions (Yang et al., 2015). Therefore, it is important to study how the psychological status of college students was influenced by the pandemic.

Psychological resilience (PR) can play an important role for college students as they adapt to changes brought about by the pandemic and seek to restore their mental health (Yi et al., 2020). PR refers to the ability or dynamic process of an individual to adapt and to develop normally after encountering a serious threat. Past studies have reported that PR can cushion the psychological trauma caused by terrorist attacks (Zeidner and Kampler, 2020), public health events (Ran et al., 2020), cancer (Mystakidou et al., 2015), depression (Howell et al., 2020), and negative emotions (Abiola and Udofia, 2011). It can also help individuals reduce their vulnerability to challenges and difficulties in the work environment (Howell et al., 2020). To date, no study related to the COVID-19 pandemic has looked specifically at the mechanism of the PR of college students as the outcome variable, and so it is important to study this phenomenon.

Many factors affect PR, including general self-efficacy (GSE). Academic research on the relationship between GSE and PR has shown a significant correlation between the two (Schumacher et al., 2005; Chen, 2020). Martin and Marsh (2006) reported that GSE has a significant predictive effect on academic resilience. However, the relationship between GSE and PR is complex, and the associated mechanisms are not yet clear. During the COVID-19 pandemic, research on GSE and PR usually addressed the psychological state of front-line medical staff and patients (Bidzan et al., 2020; Casali et al., 2020; Zhang J. et al., 2020). College students were mainly the target of descriptive research and analysis of the causes of changes in psychological status and behavior (Alemany-Arrebola et al., 2020; Ao et al., 2020; Chen et al., 2020). To date, there has been limited investigation of specific mechanisms associated with the changes in the psychological status of college students in light of this major public health event. In order to explore these mechanisms in depth, this study examined the relationship between GSE and PR of college students under the influence of the COVID-19 pandemic, as well as the mediating role of posttraumatic growth (PTG) between the two and the moderating role of deliberate rumination (DR). Further, gender was used as a control variable. In the next section, the four variables will be further defined, and the influencing variables and the relationship between them will be introduced.

## Literature Review and Hypotheses

### General Self-Efficacy

In the theory of social cognition, Bandura defined *self-efficacy* as an individual's judgment on whether they can successfully deal with internal and external environments (Bandura, 1990). Individuals' beliefs about their abilities affect their attitudes, measures, energy input, persistence, and even their ways of thinking (Bandura, 1990). Therefore, GSE can have a predictive effect on an individual's ability to adapt to changing environments. More practically, the GSE of an individual can have an impact on whether a certain goal can be successfully achieved.

Studies have shown that patients with high GSE have lower stress and greater PR during treatment and that it has a long-term impact on the resilience of a patient after treatment (Zhang W. et al., 2020), improving their quality of life (Chen et al., 2018; Hinz et al., 2019). It can be seen that a high sense of GSE can help people deal with adversity.

### Psychological Resilience

In the 1970s, psychologists found that although many of the victims of child abuse had suffered long-term repercussions, those experienced by some children were mild (Mrazek and Mrazek, 1987). Scholars named the ability or dynamic process of individuals to adapt and maintain good development after encountering serious threats as “resilience.” Rutter proposed that *protective factors* can “affect, improve or change a person's response to hazards in the environment, and these hazards may lead to poor adaptation results.” He also emphasized that protective factors are not the same as positive experiences and do not necessarily constitute a pleasant occurrence in any ordinary sense; rather, their role is to change future responses to adversity. Therefore, the protective factor can be an experience, a quality inherent in the individual, characteristics of the surrounding environment, etc. (Rutter, 1985; Mrazek and Mrazek, 1987). Since the concept of PR was first put forward, research has focused on exploring and analyzing its characteristics and the mechanisms that promote it.

The intensification of research on PR and on protective factors has led to the development of a relatively complete theoretical model. In 2000, Mandelco and Peery, summarizing previous research based on the children's PR systems, offered the Organizational Framework of Resilience (Mandleco and Peery, 2000). These models categorize protective factors as internal and external. *Internal* factors include biological factors (such as physical condition, temperament, gender) and psychological factors (such as intelligence, cognitive style, problem-solving skills, and personality). External factors include family, society, school, and peer groups.

Although the relationship between internal and external factors differs in the two models, their common core emphasizes that the support that protective factors provide to individuals facing adversity determines the existence and development of their PR. The enhancement of PR thus lies not in avoiding stress but in how people deal with life changes and how they act in the face of adversity (Rutter, 1985), and this response is based on internal and external factors. Therefore, when people face adversity, traumatic events, and after-events, their level of PR plays an important role in maintaining or recovering their mental health. Studies have shown a significant correlation and predictive effect between GSE and PR (Schumacher et al., 2005; Martin and Marsh, 2006; Chen, 2020), but no empirical study has confirmed the mechanism that operates between the two. Therefore, based on the above literature review, we propose the following hypothesis:

Hypothesis 1: College students' sense of GSE has a positive correlation with PR.

### Posttraumatic Growth

In 1996, Tedeschi and Calhoun (1996) proposed the concept of PTG, which refers to the development of positive changes after an individual experiences trauma. These positive changes may include interpersonal relationships, new possibilities, personal strength, mental state, and appreciation of life (Tedeschi and Calhoun, 2004). The occurrence of PTG can help individuals think more and take coping measures more proactively after experiencing traumatic events. However, PTG does not occur naturally. Many studies have examined the factors that predict whether individuals can achieve positive changes after trauma, showing that GSE (Blackburn and Owens, 2015; Jurisova, 2016), social support (Jia et al., 2017; Frey et al., 2019), challenging core beliefs, rumination (Ramos et al., 2018; Zhang et al., 2018), self-esteem (Ma et al., 2019), resilience (Wu et al., 2015; Yun et al., 2020), and other factors can act directly or indirectly to promote individual PTG.

Among these predictors, high GSE has been shown to promote and improve response measures and behaviors of nursing staff (Jurisova, 2016), veterans (Blackburn and Owens, 2015), and cancer patients (Yu et al., 2014; Mystakidou et al., 2015) to adversity that can lead to individual PTG. Based on the above research, it is clear that GSE has a positive predictive effect on PTG. Therefore, we propose the following hypothesis:

Hypothesis 2: In the context of the COVID-19 pandemic, the GSE of college students has a positive correlation with their PTG.

It is also clear that there is a significant correlation between PTG and PR (Kukihara et al., 2014; Wu et al., 2015; Poudel-Tandukar et al., 2019). Studies have reported that strong PR can help political refugees and survivors of natural disasters better endure trauma during the event and then recover and maintain more positive mental health state after the event (Kukihara et al., 2014; Poudel-Tandukar et al., 2019). However, little research has been done on the mechanisms that operate between PTG and PR. Tedeschi and Calhoun suggested that the growth that people achieve after experiencing traumatic events may improve their ability to “balance thinking and action, weighing the known and unknown lives.” According to these authors, such persons can better accept negative events, are more open, and can more effectively solve the obstacles and problems they meet in life (Calhoun and Tedeschi, 1999, p. 21). In theory, a person's experience and positive changes after trauma can be protective factors for the development of PR. But few studies have confirmed and analyzed the mechanism of PTG with regard to PR. Further, few studies on PTG have used public health events as the research context: Usually, research subjects are patients and veterans. Finally, there is a lack of research on the internal specific mechanism of interaction among the GSE, PR, and PTG of college students. Therefore, we offer the following hypotheses:

Hypothesis 3: In the context of the COVID-19 epidemic, the PTG of college students has a positive correlation with PR.

Hypothesis 4: In the context of the COVID-19 epidemic, the PTG of college students plays a mediating role in the relationship between GSE and PR.

### Deliberate Rumination

*Rumination* means ruminating thinking, which refers to an individual's repeated thinking about a negative event, its possible causes and consequences, and their own negative emotional state (Nolen-Hoeksema et al., 2008). After reviewing the research on whether the results of rumination are constructive and whether its impact is positive or negative, Cann et al. (2011) divided rumination into DR and intrusive rumination. Intrusive rumination is an uninvited reflection in the individual's self-perceived world, and the content of the reflection is usually negative. DR refers to consciously thinking positively about traumatic events and even about solutions, and research on this form of rumination has focused on its relationship with posttraumatic stress disorder and with PTG. It has been found to be an important predictor of PTG after natural disasters (Zhou and Wu, 2015), special work experience (Yang and Ha, 2019), cancer treatment (Mundey et al., 2019), bereavement (Taku et al., 2008), and other traumatic events. It can also mediate the relationship between intrusive rumination and PTG (Zhou et al., 2015). However, there is little research on the relationship between DR and GSE.

General self-efficacy can affect cognition, motivation, emotion, and selection process differently (Bandura, 1990). When people have a low sense of GSE, that is, a low degree of mastery of events and long-term psychological tension, they feel powerless and unable to solve the problem. Under negative cognitive conditions, they are more likely to produce intrusive ruminations, which can exacerbate negative emotions or depression (Nolen-Hoeksema et al., 1999) or posttraumatic stress disorder (Zhou and Wu, 2016). When people have a higher sense of GSE, they are more motivated and more active to understand a problem, reflect on the problem to achieve DR in the face of adversity (Andersson et al., 2014). Therefore, we offer the following hypothesis:

Hypothesis 5: General self-efficacy has a positive correlation with DR.

The type of rumination with which individuals respond to a traumatic event will affect their psychological development thereafter. DR can predict the occurrence of individual PTG, while intrusive rumination can predict the occurrence of individual posttraumatic stress disorder (Zhou et al., 2015; Zhou and Wu, 2016). DR can help an individual incorporate traumatic experiences into their own cognitive model and reconstruct their understanding of their self and so promote PTG. Other studies have shown that DR can regulate the relationship between intrusive rumination and PTG and that it can contribute to PTG in traumatized adults (Li et al., 2018; Kim and Bae, 2019). In addition to Hypothesis 5, we propose the following hypotheses:

Hypothesis 6: Deliberate rumination has a positive correlation with PTG.

Hypothesis 7: The interaction of GSE and DR has a positive correlation with PTG.

The positive changes brought about by PTG have multiple dimensions. Studies have shown that these positive changes include a better view of life (Affleck et al., 1985), a greater appreciation for life (Joseph et al., 1993), a new view of the priority of events (Tedeschi and Calhoun, 1996), etc. These positive changes incline people to think positively when ruminating and to take active countermeasures (Zhou and Wu, 2015). DR can cause people to reflect on and summarize the causes, significance, and personal factors related to the incident. These reflections and summaries can add to the experience they bring in dealing with future adversity and become a protective factor. Therefore, we offer the following hypotheses:

Hypothesis 8: Posttraumatic growth has a positive correlation with DR.

Hypothesis 9: Deliberate rumination has a positive correlation with PR.

Hypothesis 10: The interaction between PTG and DR has a positive correlation with PR.

**Figure 1:**
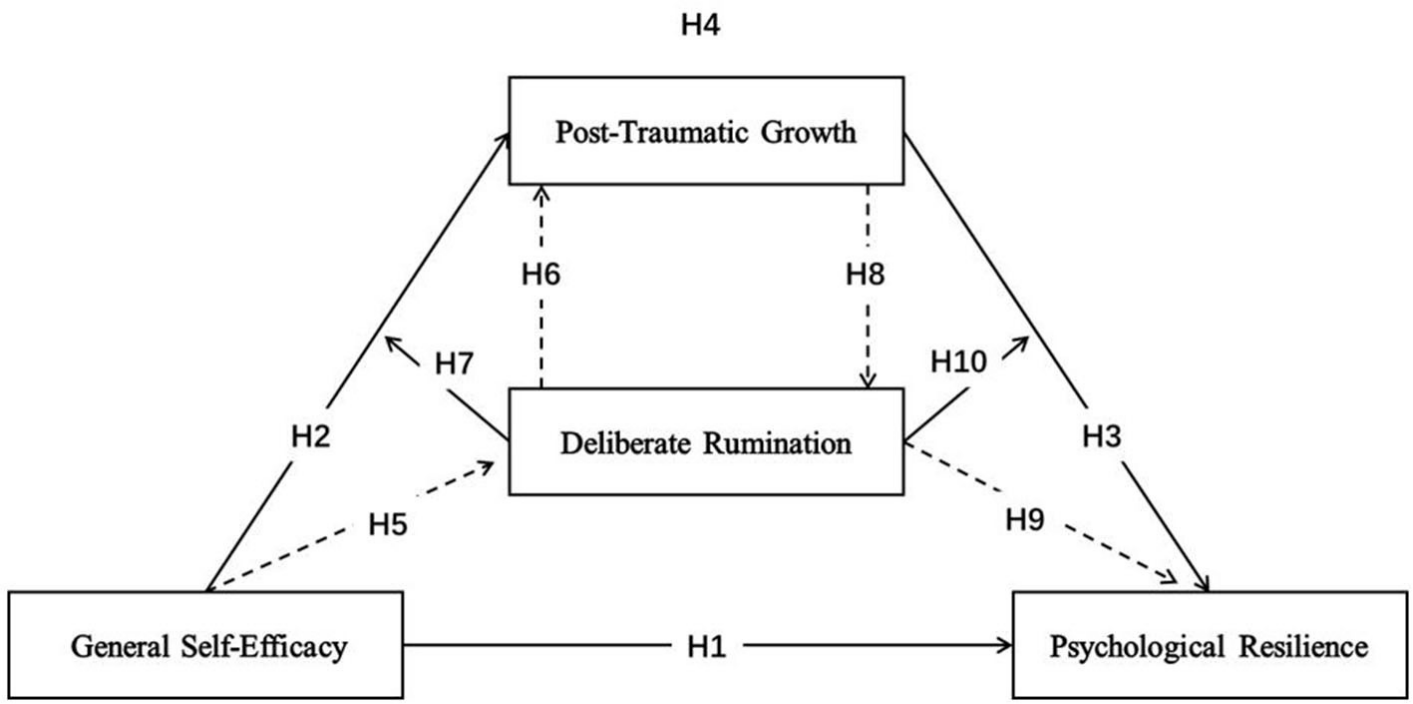
The relationships examined in the study. The solid lines represent the direct assumptions of the moderated mediation model. Dotted lines represent supporting assumptions that support mediating and moderating effects.

Figure 1 shows a diagram of the moderated mediation model proposed in the 10 hypotheses, which depicts the relations between the independent, mediator, moderator, and dependent variables.

## Materials and Methods

### Participants and Procedures

This study was conducted in an independent college in Guangdong Province, China. The college offers 44 undergraduate professional courses to more than 20,000 students. Of these, 918 students participated in the research and filled out an online questionnaire. Considering that during the epidemic, people from Guangdong Province were more severely affected than people from other provinces in the sample, permanent addresses other than Guangdong province were eliminated. After invalid samples, outliers, and questionnaires with permanent addresses other than Guangdong Province from the obtained data were excluded, the sample size was reduced to 881. Among the interviewees, the number of males was 317 (35.982%) and the number of females was 564 (64.018%), all from the same grade and of similar age. In order to gather research ideas and potential hypotheses, we conducted an exploratory focus group interview at the college before finalizing the research design. Most of the interviewees stated that they had experienced anxiety, depression, and stress during the COVID-19 pandemic. During the lockdown period, their life patterns, learning styles, family relationships, and personal psychological status were all affected to varying degrees.

The survey questionnaire was conducted between April 10, 2020 and June 15, 2020. During recess time, the researchers sent the code for the questionnaire to college students. Those who wished to participate scanned the QR code and filled out the questionnaire on their smartphones. A QR code, or quick response code, is a matrix barcode that can store information and jump to a web page. In China, the use of QR codes is very common, and main functions include payment, anti-counterfeiting, and the opening of specific websites on smartphones. Using a QR code to link to the questionnaire made it convenient for students to complete and later for the researchers to collect and analyze the data. Before a student completed the questionnaire, we explained to them the content of the research and obtained their consent.

### Materials

The questionnaire used in this study consisted of five sections: (a) demographic information, (b) Psychological Resilience Scale, (c) General Self-Efficacy Scale, (d) Posttraumatic Growth Inventory, and (e) Event-Related Rumination Inventory. The demographic information included gender, address, and profession. The General Self-Efficacy Scale, the Posttraumatic Growth Inventory, and the Event-Related Rumination Inventory had been originally developed in English and were translated into Chinese for use in this study. In order to improve the accuracy of the translations, a back-translation method was adopted (Brislin, 1970). The first translator translated the original English scale into Chinese. A second translator translated the translated scale back into English, and then a third translator combined the original, translated, and back-translated scales. The three versions of the scale were then compared, and the translation was revised to ensure the equivalence of the questionnaire content. The Psychological Resilience Scale was available in Chinese and so was used in the study without change.

#### General Self-Efficacy Scale

The General Self-Efficacy Scale developed by Schwarzer et al. (1997) was used to measure participants' GSE. The initial version of the scale had a total of 20 items but was subsequently revised to 10 items in 1997. After discussion, we deleted three of these, leaving seven items. All items were scored on a four-point Likert scale, ranging from 1 (completely incorrect) to 4 (completely correct). The theoretical score ranges from 7 to 28. The higher the score, the higher the participant's level of GSE. In this study, the Cronbach's alpha for this scale was 0.875.

#### Psychological Resilience Scale

This study uses the Psychological Resilience Scale proposed by Hu and Gan (2008) after aggregating relevant domestic and foreign studies (Connor and Davidson, 2003; Hu and Gan, 2008). The scale has 27 items, divided into five dimensions, namely, goal focus, emotional control, positive cognition, family support, and interpersonal assistance. Respondents were asked to use a five-point Likert scale scoring method to express their approval of each item, ranging from 1 (completely true) to 5 (completely untrue). The theoretical score ranges from 27 to 135. The higher the score, the stronger the PR of the participant. In this study, the Cronbach's alpha for this scale was 0.860.

#### Posttraumatic Growth Inventory

This study uses the Chinese version of the Posttraumatic Growth Inventory originally proposed by Tedeschi and Calhoun (1996) and translated and adapted by Geng et al. (2011) to suit the Chinese context (Geng et al., 2011). The 21-item scale is divided into five dimensions, namely, interpersonal relationships, new possibilities, personal strength, spiritual changes, and appreciation of life. The scale uses the six-point Likert scale scoring method, and the scoring ranges from 1 (feeling no change) to 6 (feeling a lot of change). The theoretical score ranges from 21 to 126. The higher the score, the higher the level of PTG. In this study, Cronbach's alpha was 0.958.

#### Event-Related Rumination Inventory

To measure the DR of students, this study adopted the Event-Related Rumination Inventory developed by Cann et al. (2011). The full scale consists of 20 items, 10 each deliberative rumination and intrusive rumination, and uses a four-point Likert scale scoring method, ranging from 1 (never) to 4 (always). In this study, only the deliberative rumination items were used, so the theoretical score ranges from 10 to 40. The higher the score, the higher the frequency of deliberative rumination. In this study, Cronbach's alpha was 0.913.

### Data Analysis

SPSS 23.0 and SPSS plug-in PROCESS (version 3.3) were used to analyze the collected data. First, a descriptive analysis of the scores of GSE, PR, PTG, and DR scales was carried out. Second, the Pearson product–moment correlation coefficient was used to evaluate the correlation between continuous variables. Finally, the SPSS plug-in PROCESS (version 3.3) was used to test the mediating role of PTG and the moderating role of DR in the relationship between GSE and PR. The PROCESS plug-in was developed by Hayes (2013) and Bolin (2014) pacifically for path analysis-based conditioning and mediation analysis and their combinations.

## Results

SPSS 23.0 was used to calculate the average, SD, and correlation coefficients of the four variables of GSE, PR, PTG, and DR. The results of the analysis are shown in Table 1.

**Table 1:** Means, SDs, and correlations of variables (*N* = 881).

	**M**	**SD**	**1**	**2**	**3**	**4**
1. General self-efficacy	2.318	0.537	1			
2. Psychological resilience	3.417	0.443	0.267^**^	1		
3. Posttraumatic growth	3.301	1.003	0.466^**^	0.414^**^	1	
4. Deliberate rumination	2.013	0.520	0.216^**^	0.078^*^	0.353^**^	1

* *p < 0.05*,

** *p < 0.01*.

The results showed that GSE was significantly and positively correlated with DR, PR, and PTG (*r* = 0.216, *p* < 0.01; *r* = 0.267, *p* < 0.01; *r* = 0.466, *p* < 0.01). PR was significantly and positively correlated with DR and PTG (*r* = 0.078, *p* < 0.05; *r* = 0.414, *p* < 0.01). In addition, PTG and DR were significantly and positively correlated (*r* = 0.353, *p* < 0.01).

### Mediation Analysis

SPSS plug-in PROCESS (version 3.3) Model 4 was used for this analysis. Gender was used as a control variable. As shown in Table 2, GSE can significantly and positively predict PR and PTG (*B* = 0.097, *SE* = 0.029, *t* = 3.356, *p* < 0.01; *B* = 0.895, *SE* = 0.057, *t* = 15.82, *p* < 0.001), and PTG can significantly and positively predict PR (*B* = 0.16, *SE* = 0.015, *t* = 10.494, *p* < 0.001). Therefore, PTG plays a mediating role between GSE and PR.

**Table 2:** Regression results for mediation analysis (*N* = 881).

**Outcome**	**Predictors**	**B**	**SE**	***t***	**LLCI**	**ULCI**
Posttraumatic growth	Constant	0.989	0.184	5.367^***^	0.628	1.351
	Gender	0.144	0.063	2.282^*^	0.020	0.269
	GSE	0.895	0.057	15.82^***^	0.784	1.006
	*R*^2^ = 0.222, *F* = 125.388, *p* < 0.001					
Psychological resilience	Constant	2.504	0.085	29.602^***^	2.338	2.67
	Gender	0.098	0.029	3.404^**^	0.041	0.154
	GSE	0.097	0.029	3.356^**^	0.040	0.154
	PTG	0.160	0.015	10.494^***^	0.130	0.19
	*R*^2^ = 0.189, *F* = 68.293, *p* < 0.001					

*LLCI, Boot CI lower limit; ULCI, Boot CI upper limit*.

* *p < 0.05*,

** *p < 0.01*,

*** *p < 0.001*.

In addition, bootstrap analysis results showed that the direct effect and indirect effect between GSE and PR do not contain 0 between the upper and lower limits of the 95% bootstrap confidence interval (95% CI = [0.040, 0.154], 95% CI = [0.784, 1.006], 95% CI = [0.130, 0.190]). This showed that after controlling for gender variables, PTG as a mediating variable plays a significant role in Model 4; that is, PTG plays the part of a mediating role between GSE and PR. Moreover, the mediating effect of PTG accounted for 59.583% (see Table 3).

**Table 3:** Direct and indirect effects of GSE on PR (*N* = 881).

**Effect**	**B**	**Boot SE**	**Boot LLCI**	**Boot ULCI**	**Relative effect size**
Total effect	0.240	0.027	0.187	0.293	–
Direct effect	0.097	0.029	0.040	0.154	40.416%
Indirect effect	0.143	0.019	0.107	0.181	59.583%

*Boot LLCI, Boot CI lower limit; Boot ULCI, Boot CI upper limit*.

### Mediating Moderation Analysis

On the basis of the significant mediating effect of PTG, DR was added as a moderating variable. Model 58 continued to be used for analysis. The results showed that the interaction between GSE and DR had a negative effect on PTG (*B* = −0.176, *SE* = 0.088, *t* = −1.992, *p* < 0.05). The interaction of PTG and DR had a positive effect on PR (*B* = 0.051, *SE* = 0.022, *t* = 2.343, *p* < 0.05) (see Table 4).

**Table 4:** Regression results for moderation analysis (*N* = 881).

**Outcome**	**Predictors**	**B**	**SE**	***t***	**LLCI**	**ULCI**
Posttraumatic growth	Constant	−0.11	0.105	−1.046	−0.315	0.096
	Gender	0.073	0.061	1.198	−0.047	0.193
	GSE	0.776	0.056	13.912^***^	0.666	0.885
	DR	0.532	0.059	9.054^***^	0.417	0.648
	GSE^*^DR	−0.176	0.088	−1.992^*^	−0.348	−0.003
	*R*^2^ = 0.289, *F* = 88.956^***^, *p* < 0.001					
Psychological resilience	Constant	3.233	0.049	65.979^***^	3.137	3.329
	Gender	0.107	0.029	3.73^***^	0.051	0.163
	GSE	0.103	0.029	3.572^***^	0.046	0.16
	DR	−0.09	0.028	−3.234^**^	−0.145	−0.036
	PTG	0.175	0.016	11.065^***^	0.144	0.206
	PTG^*^DR	0.051	0.022	2.343^*^	0.008	0.093
	*R*^2^ = 0.202, *F* = 44.393^***^, *p* < 0.001					

*GSE, general self-efficacy; PTG, posttraumatic growth; DR, deliberate rumination*.

* *p < 0.05*;

** *p < 0.01*;

*** *p < 0.001*.

**Figure 2:**
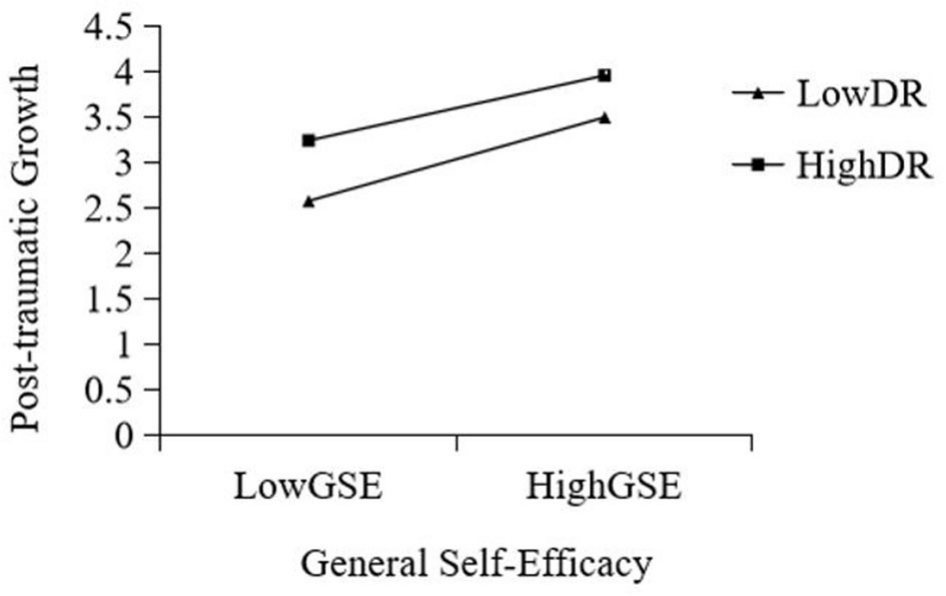
Interactive effects of GSE and DR on PTG. DR, deliberate rumination; GSE, general self-efficacy.

**Figure 3:**
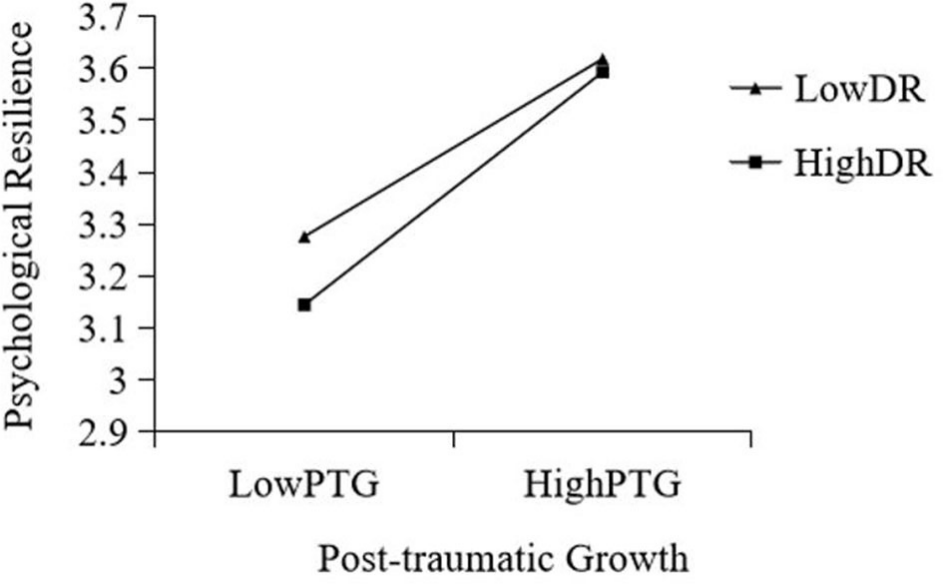
Interactive effects of PTG and DR on PR. DR, deliberate rumination; PTG, posttraumatic growth.

The moderating effect of DR was further analyzed using a simple slope test. The adjustment variables were grouped according to the mean score of DR plus or minus one SD. The mean plus one SD was designated the high DR group, and the mean minus one SD was designated the low DR group (see Table 5). The analysis yielded the following findings:

- When the level of DR was low, as the level of GSE increased, the level of PTG of college students showed a significant upward trend (Effect = 0.867, *t* = 12.183, *p* < 0.001).
- When the level of DR was high, as the level of GSE increased, the level of PTG of college students showed a significant decrease (Effect = 0.684, *t* = 9.315, *p* < 0.001).
- When the level of DR was low, with the increase in PTG, the level of resilience PR of college students increased significantly (Effect = 0.149, *t* = 7.823, *p* < 0.001).
- When the level of DR was high, with the improvement of PTG, the level of PR of college students increased significantly (Effect = 0.201, *t* = 10.161, *p* < 0.001). This shows that the influence of PTG on PR increases with an increase in DR (see Figures 2, 3).

**Table 5:** The mediating response value of independent variables GSE and PTG at different levels of the modulating variable DR (*N* = 881).

**Predictor**	**DR**	**Effect**	***t***	**Boot SE**	**Bootstrap 95% CI**	
					**LLCI**	**ULCI**
General self-efficacy	−0.520	0.867	12.242^***^	0.071	0.728	1.006
	0.000	0.776	13.912^***^	0.056	0.666	0.885
	0.520	0.684	9.315^***^	0.073	0.540	0.829
Posttraumatic growth	−0.520	0.149	7.823^***^	0.019	0.111	0.186
	0.000	0.175	11.065^***^	0.016	0.144	0.206
	0.520	0.201	10.161^***^	0.020	0.162	0.24

*GSE, general self-efficacy; PTG, posttraumatic growth; DR, deliberate rumination*.

*** *p < 0.001*.

**Figure 4:**
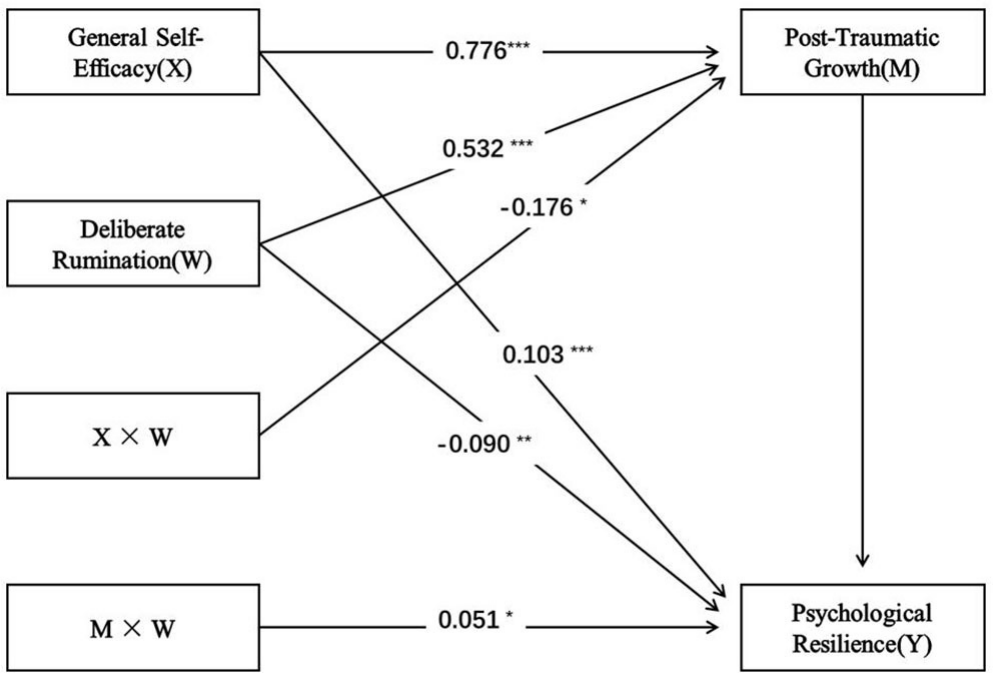
The relationships between general self-efficacy, psychological resilience, posttraumatic growth and deliberate rumination. ^*^p < 0.05; ^**^p < 0.01; ^***^p < 0.001.

The above results showed that after controlling for gender variables, DR as a moderating variable in Model 58 played a significant role in regulating the relationship between GSE and PTG, and between PTG and PR. The model is shown in Figure 4.

## Discussion

This study focuses on the relationship and the related mechanisms between GSE and PR during the COVID-19 pandemic, the mediating role of PTG, and the moderating role of DR. The results of the study show that all four variables are positively correlated. GSE has a direct predictive effect on DR and PTG mediating between the two. At the same time, DR hinders the effect of GSE on PTG but enhances the positive effect of PTG on PR.

### Discussion of the Results

First, the study results are basically consistent with Hypothesis 1 and with previous research, in which a positive relationship between GSE and PR was found (Martin and Marsh, 2006; Sanchez-Teruel and Robles-Bello, 2020). As Sánchez-Teruel et al. maintained in their study of the PR of young Moroccan immigrants, GSE is one of the most predictable and positive factors affecting PR (Sanchez-Teruel and Robles-Bello, 2020). Similarly, Martin et al. proposed in their study of academic resilience that academic self-efficacy is an important predictor of academic resilience (Martin and Marsh, 2006).

Second, the results of this study are consistent with Hypotheses 2, 3, and 4 and with previous studies, confirming that PTG can partially and positively mediate the relationship between GSE and PR. This means that the effect of college students' sense of GSE on their PR is partly produced through PTG. As Tedeschi and Calhoun (2004) stated, some individuals or groups recover from traumatic events better than others, and the focus should be not on the period of psychological rebound or recovery but rather on the abilities and resources possessed before the event. Similarly, Lagadec pointed out that the ability to respond to a crisis largely depends on the structure that existed before it occurred (Lagadec and Phelps, 1991). Masten maintained that individuals with a brain in good working condition have the ability to learn and acquire the ability to cope with problems and that this learned adjustment ability can help them cope with a variety of threats (Southwick et al., 2014). Moreover, when individuals have established close relationships with others, these relationships can provide a deep sense of security and social support, and people are able to enhance their own PR in these relationships. In summary, traumatic events have caused individuals to undergo positive changes and improvement in interpersonal relationships, future possibilities, personal strength, mental state, and attitudes toward life. Those with high GSE have stronger beliefs and ability for action when dealing with adversity. This solid foundation, coupled with positive improvements after trauma, can provide them with coping strategies and support if they again face adversity, increasing their physical and psychological resistance to negative effects: in other words, enhancing individual PR.

Third, the results of this study are consistent with Hypothesis 5, confirming that GSE has a positive predictive effect on DR. There are few studies on the role of GSE in DR. From the perspective of self-efficacy theory, GSE refers to an individual's belief that they will behave appropriately in the face of adversity (Bandura, 1990). A person who believes that they have sufficient control over events can be expected to deal with such events more actively and proactively in real life. Studies have shown that self-efficacy is significantly related to positive coping. The higher the GSE of college students, the more likely they are to adopt optimistic and positive thinking and behaviors when coping with adversity (Tong, 2004). Moreover, high GSE can prevent the negative effects of emotion-oriented coping (Brands et al., 2014). Therefore, we suggest that when reflecting back on events, individuals with high GSE may be more inclined to ruminate deliberately rather than intrusively.

Fourth, the results of this study are consistent with Hypothesis 6 and previous studies, confirming that DR can help individuals think positively after a traumatic event and find positive meaning in it. Several relevant studies have confirmed that DR is an important factor in predicting PTG and that it can also promote PTG by mediating intrusive rumination (Affleck et al., 1985; Taku et al., 2009; Stockton et al., 2011).

Fifth, the results of this study failed to confirm Hypothesis 7. Studies have shown that DR can have a negative impact on the relationship between GSE and PTG: If college students had a higher level of DR, a high sense of GSE was more likely to reduce the possibility of positive changes after trauma. Zhou and Wu, who carried out a longitudinal study on PTG and posttraumatic stress disorder, concluded that DR has a positive effect on PTG in the short term but may have a negative effect over the long term due to repeated exposure to the traumatic event (Zhou and Wu, 2016). Therefore, we suggest that the results of study may have been influenced by the time span of the subjects experienced COVID-19.

Sixth, the results of this study are consistent with the results of Hypotheses 8, 9, and 10 and previous studies. DR can have a positive impact on the relationship between PTG and PR. This indicates that college students with a higher level of DR can improve their PR effectively through positive changes that occur after they experience a traumatic event. The relation between PTG and PR, stated in H3, has been affected by DR. DR can deepen the positive changes after trauma, enabling individuals to achieve a better and faster psychological rebound and recovery.

### Implications

Theoretically, this research links GSE and PR, a result not seen previously. This study has increased understanding of the impact of GSE on the PR of college students in the face of a public health emergency. In addition, the results of using PTG as a mediating variable and DR as a moderating variable show that in a public health emergency, PTG of college students can enhance their PR. Although DR had a positive effect on the PTG of college students, DR had a negative regulatory effect on the relationship between GSE and PTG.

From a practical perspective, knowledge of the relationship between the four variables examined in this study may further the understanding of the PR improvement mechanism of college students and even the general public. We recommend that college students engage in DR after experiencing a traumatic public health event. By doing this, they could promote PTG and improve their PR, thus preparing themselves psychologically to respond to subsequent similar events.

### Limitations and Future Research

This study has three limitations. The first limitation lies in the sampling. Participants in this study were from a single year at a single university; the specific majors and socioeconomic status of the participants were not investigated in detail. Therefore, the results do not account for differences between college students of different majors, different grades, and different socioeconomic status. Therefore, follow-up research could extend the sample to other colleges and universities from different provinces and could distinguish between students with different majors.

The second limitation of this study is the use of a cross-sectional research design. The data and results obtained from the questionnaire can only reflect the psychological conditions and characteristics of college students over the short term, and so it is difficult to dynamically and comprehensively depict the relationship between GSE and PR. Therefore, in future research, we will try a longitudinal design so as to better explore the continuous and long-term interaction between the variables.

The third limitation of this study is that the data relied solely on self-reported measurements. Although the self-reported questionnaires can help us understand the students' perception of their psychological status, this method may lead to the exaggeration of study associations due to common method variance and social desirability (Podsakoff et al., 2003). Therefore, in subsequent research, the relevance of the current model can be further confirmed by combining a multi-information approach, such as parents' or teachers' rating data on students' psychological conditions (Ma et al., 2020).

## Conclusion

This study explored the relationship between GSE and PR, the mediating role of PTG, and the moderating role of DR. The results show that, in response to a public health emergency, college students with high GSE can improve their PR under the mediating influence of PTG. Further, DR can negatively regulate the relationship between GSE and PTG and positively regulate the relationship between PTG and PR.

## Data Availability Statement

The raw data supporting the conclusions of this article will be made available by the authors, without undue reservation.

## Ethics Statement

The studies involving human participants were reviewed and approved by Ethics Committee of South China Normal University. The patients/participants provided their written informed consent to participate in this study.

## Author Contributions

WZ and YX designed the research and reviewed and edited the paper. XW, WZ, YX, and JW carried out the literature search and data analysis. WZ, XW, YX, JW, YZ, JS, DH, and ZZ wrote the paper. All authors have read and agreed to the published version of the manuscript.

## Conflict of Interest

The authors declare that the research was conducted in the absence of any commercial or financial relationships that could be construed as a potential conflict of interest.
